# A graphical user interface for RAId, a knowledge integrated proteomics analysis suite with accurate statistics

**DOI:** 10.1186/s13104-018-3289-6

**Published:** 2018-03-15

**Authors:** Brendan Joyce, Danny Lee, Alex Rubio, Aleksey Ogurtsov, Gelio Alves, Yi-Kuo Yu

**Affiliations:** National Center for Biotechnology Information, National Library of Medicine, National Institutes of Health, 8600 Rockville Pike, Bethesda, MD 20894 USA

**Keywords:** Peptide identification, Protein identification, Proteomics, Tandem mass spectrometry

## Abstract

**Objective:**

RAId is a software package that has been actively developed for the past 10 years for computationally and visually analyzing MS/MS data. Founded on rigorous statistical methods, RAId’s core program computes accurate *E*-values for peptides and proteins identified during database searches. Making this robust tool readily accessible for the proteomics community by developing a graphical user interface (GUI) is our main goal here.

**Results:**

We have constructed a graphical user interface to facilitate the use of RAId on users’ local machines. Written in Java, RAId_GUI not only makes easy executions of RAId but also provides tools for data/spectra visualization, MS-product analysis, molecular isotopic distribution analysis, and graphing the retrieval versus the proportion of false discoveries. The results viewer displays and allows the users to download the analyses results. Both the knowledge-integrated organismal databases and the code package (containing source code, the graphical user interface, and a user manual) are available for download at https://www.ncbi.nlm.nih.gov/CBBresearch/Yu/downloads/raid.html.

**Electronic supplementary material:**

The online version of this article (10.1186/s13104-018-3289-6) contains supplementary material, which is available to authorized users.

## Introduction

RAId is a software package for identifying peptides and proteins via analyzing tandem mass spectrometry data. An important feature of RAId is its use of spectrum-specific statistics [1] for inferring peptide *P*- and *E*-values. Based on the extension of the central limit theorem (CLT), RAId’s score distribution can be derived theoretically [2] with one parameter for fitting the skewness of the corresponding score histogram *per spectrum*. To extend accurate statistics to more scoring functions, we have also developed via dynamic programming the “all possible peptide” (APP) statistics [3, 4] which is applicable to any scoring function that sums independent contributions, including popular scoring functions such as XCorr [5], RAId score [2], Hyperscore [6], and Kscore [7]. To go beyond scoring functions that are sums of independent contributions, we have further implemented in RAId the extreme value distribution (EVD) based method for peptide significance assignment [8].

The most substantial extension of the RAId package, however, is its protein identification with accurate protein level *P*- and *E*-values. This new feature is based on our recently developed formalism for combining [9] weighted *P*-values [10] and a rigorous statistical method for protein identification [11].

In addition to the in-house developed knowledge-integrated organismal protein databases, RAId can search custom protein databases provided by the users. Our knowledge-integrated databases currently contain 20 organisms and include annotated information for single amino-acid polymorphisms (SAPs), post-translational modifications (PTMs) and their disease associations, when available. The format of the databases [12] allows for efficient peptide search with on-the-fly scope expansion to include annotated SAPs and PTMs. Users also have the option for including novel PTMs during searches.

To simplify the access to the full functionality of RAId and to integrate with RAId useful visualization and graphic tools, we have developed the *first* GUI for RAId. Although there exist other freely available proteomics analysis GUI such as OMSSAGUI [13] and SearchGUI [14], RAId_GUI is the only one that provides not only peptide/protein identifications with accurate statistics but also a large collection of additional functionalities.

## Main text

To run RAId_GUI on the Linux Operating System, the user has to download the compressed RAId software package and uncompress the archive to a local directory. Running RAId_GUI requires the X Window System (X11).

### MS/MS dataset

To demonstrate the utility of RAId_GUI we have used a dataset composed of three MS/MS files: sample one (S-1) PSM1027_07FEB15_ABRF_FT_100a.mzXML, S-2 PSM1028_07FEB15_ABRF_FT_100b.mzXML and S-3 PSM1029_07FEB15_ABRF_FT_100c.mzXML downloaded from https://www.ncbi.nlm.nih.gov/peptidome/PSE108/. This dataset is a triplicate run of a mixture composed of 49 known human proteins (Sigma-Aldrich 49). The tryptic peptides were separated via revere phase high-performance liquid chromatography using a 45 min gradient and MS/MS spectra were acquired on an LTQ Orbitrap (Thermo Electron) mass spectrometer. The Sigma-Aldrich 49 protein mixture, now referred to as Universal Proteomics Standard 1 (UPS-1), was originally designed to evaluate a method’s performance in identifying the components of a known complex protein mixture.

### Setting parameters

The command line to start RAId_GUI is:

$ java -jar RAId_GUI.jar

**Fig. 1:**
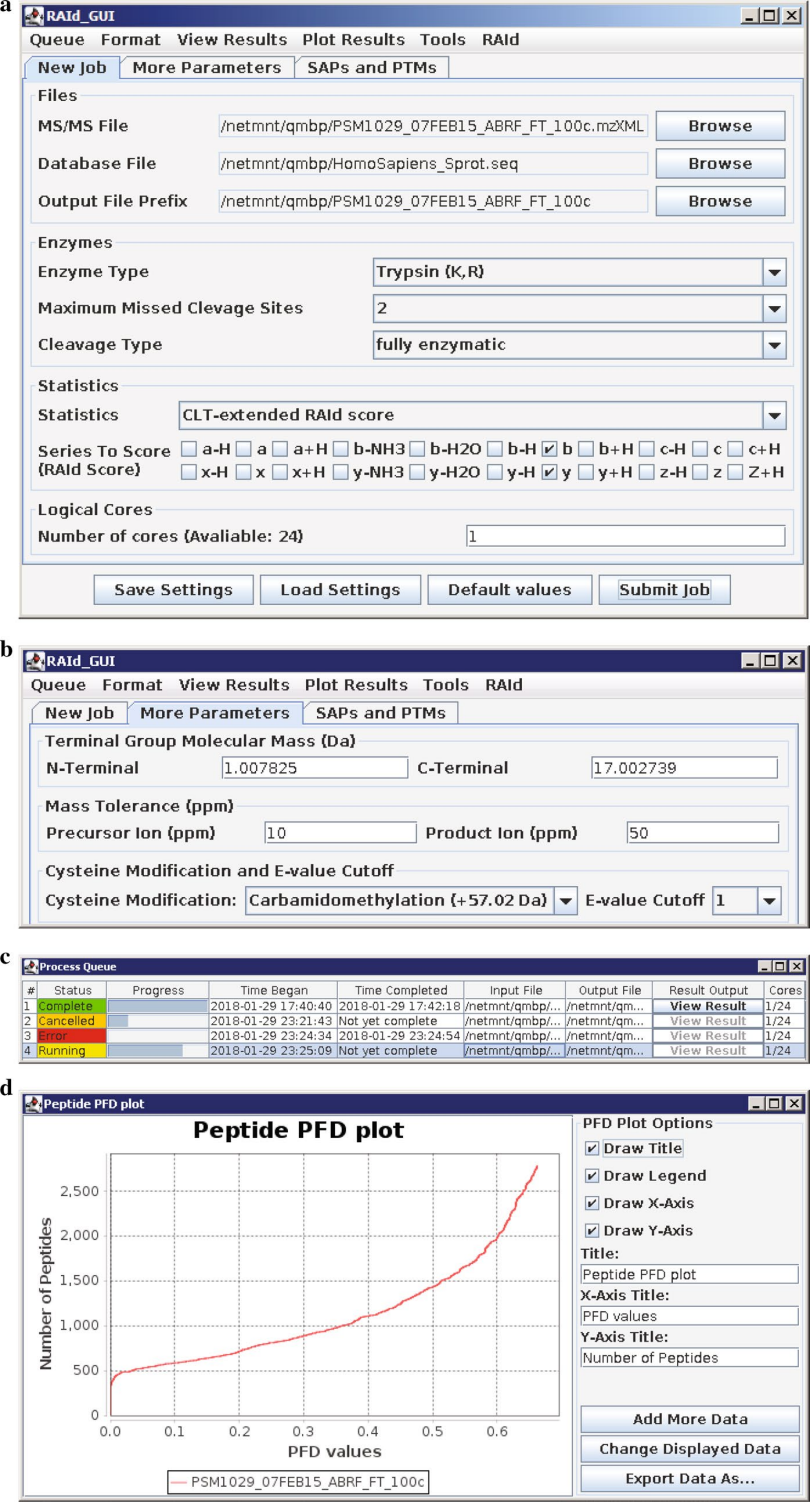
Illustrations of RAId_GUI when analyzing the spectral data file PSM1029_07FEB15_ABRF_FT_100c.mzXML. ** a** Main dialog window of RAId_GUI with appropriate parameters selected in the “New Job” tab; ** b** main dialog window of the “More Parameters” tab; ** c** the Process Queue window; ** d** peptide PFD plot, readily accessible under “Plot Results” menu, of this example run

After this command the main dialogue window appears. In the main dialogue window, there are three main tabs allowing users to set desirable search parameters. Under the New Job tab, in the Files group, the supported formats for the input MS/MS File include .dta, .pkl, .mgf, .mzData, .mzXML, .mzML, and .raw. And for our case, we shall select S-3 as our example run. As for the database, the *H. sapiens* database, *HomoSapiens_Sprot.fasta*, containing 161,521 protein sequences downloaded from http://www.uniprot.org (January 1, 2018). The user may choose his/her own Output File Prefix; we put in *PSM1029_07FEB15_ABRF_FT_100c* as an example. See the Files group of Fig. 1 a.

**Fig. 2:**
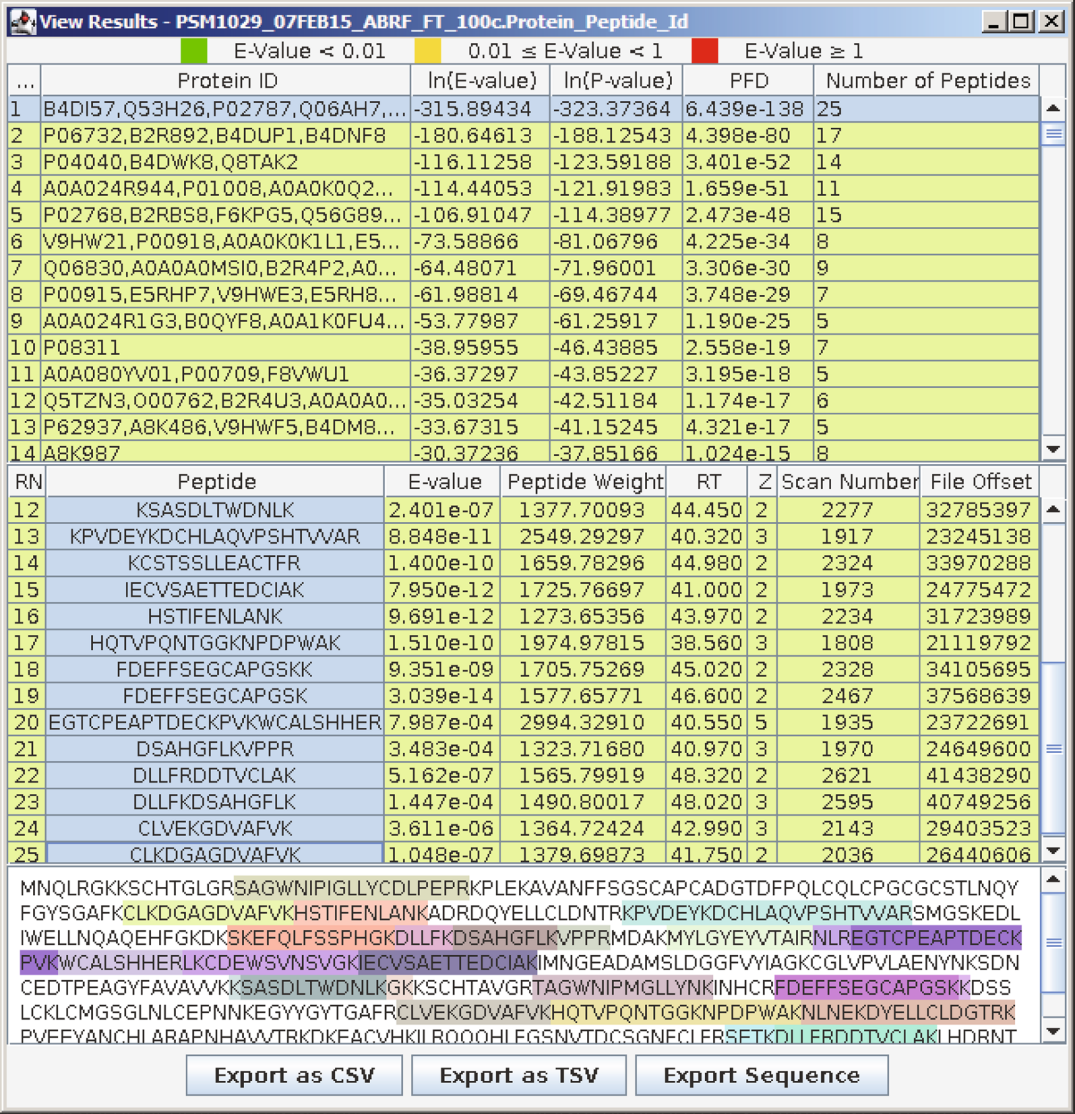
Illustrations of RAId_GUI when analyzing the spectral data file PSM1029_07FEB15_ABRF_FT_100c.mzXML. The proteins and peptides viewer shown here, available under the “View Results” menu, displays proteins and peptides identified. By selecting (highlighting) a protein, in this example the top-ranking protein, all identified peptides corresponding to that protein are shown in the middle sector of the window. Peptides selected (highlighted) in the middle sector of the window are then displayed in color at the bottom window where the full protein sequence is displayed

In general, the full knowledge-integrated organismal databases can be downloaded for use from the website provided. In addition, users may also construct a custom database that is made of protein sequences of their choice via the Format option on the menu bar by first browsing to the location of the sequence file (in FASTA).

To set the enzymatic digestion preference, users need to specify three parameters in the Enzymes group. For the example data set, Enzyme Type is *Trypsin*, maximum missed cleavage sites is *2*, and Cleavage Type is *fully enzymatic*. See the Enzymes group of Fig. 1a.

**Fig. 3:**
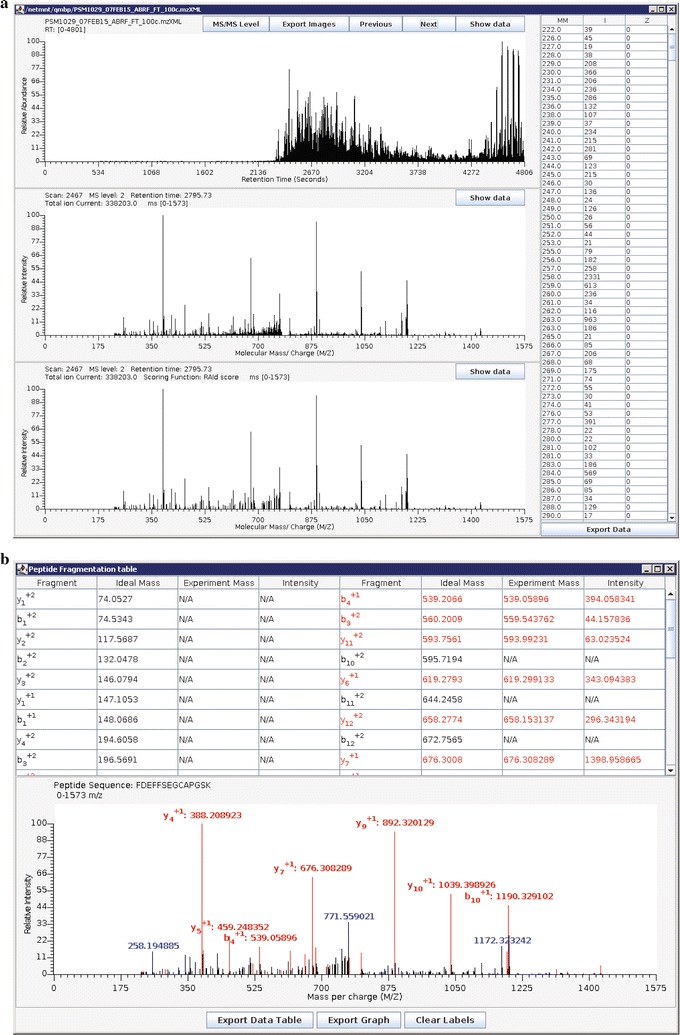
Illustrations of RAId_GUI “Tools” when analyzing the spectral data file PSM1029_07FEB15_ABRF_FT_100c.mzXML. The top sector spectra viewer in ** a** displays the chromatogram of PSM1029_07FEB15_ABRF_FT_100c.mzXML; the middle sector displays the MS/MS spectrum (Scan Number 2467) of FDEFFSEGCAPGSK, the peptide of record number 19 belonging to protein P02787 (see Fig. 2); the bottom sector displays the same spectrum filtered by the RAId score scoring function. **b** The “Peptide Product-Ions” tool using the same peptide FDEFFSEGCAPGSK and its corresponding spectrum. The top sector tabulates the theoretical fragments of the peptide with experimentally supported matches shown in red. The bottom sector shows the filtered spectrum; strong peaks having (no) theoretical matches displayed in red (blue)

In the Statistics group, combinations of several scoring functions and statistical methods are available. For this example run, we choose RAId score (as the scoring function), the *CLT-extended* statistics, and the $b-$ and $y-$ fragmentation series to score. See the Statistics group of Fig. 1a. In the Logical Cores group, users may specify the number of CPU cores to be allocated for the job. For this example run, we select *1* core. See the Logical Cores group of Fig. 1a.

To proceed, under the more parameters tab, we use the default molecular masses for N- and C-termini, *carbamidomethylation (+57.02 * Da) for cysteine modification, *10* and *50* ppm for precursor and product ion mass tolerances, and *1* for *E*-value cut-off. Under the SAPs and PTMs tab, users may set their preference for SAP and PTM inclusion during searches. For this example run, we did not include SAPs or PTMs during searches.

To avoid repetition and save time, a user may save search parameters to a file by clicking on the Save Settings button and then load it for later use by clicking on the Load Settings button. More details about search parameter options can be found in the user manual, which can be easily accessed by selecting on the menu bar the option RAId/User Guide.

### Monitoring jobs

After specifying parameters, the user can submit a job to run and a Process Queue window will open. The Process Queue window reports the status of submitted jobs. In our example run, the user will see *PSM1029_07FEB15_ABRF_FT_100c* appear as the output file. See Fig. 1c. If the Process Queue window is accidentally closed, one may access it by selecting the Queue/Process Queue option in the menu bar. When the specified number of cores is greater than the cores currently available, the Process Queue puts the job on hold until enough CPUs are free. Users may cancel/restart/remove a job by right clicking on the colored cell under status.

### Results

After a RAId job is finished, users can view the protein and peptide ID results by pressing the View Results button either in the Process Queue window or in the main menu. Users then must select any of the three types of result files (peptide ID, protein ID, protein peptide ID) for viewing. Figure 2 displays the peptide/protein ID result of the current example run. On top, one first finds a list of identified proteins. By clicking on an identified protein, that protein sequence and all the identified peptides mappable to that protein are shown. If one then clicks on the identified peptides, the corresponding segments in the protein will be highlighted in colors. This protein coverage picture can be exported by clicking on the Export Sequence button. One may also choose to export the identification results into a comma or tab separated file within which each identified protein and its corresponding peptides are displayed.

Since RAId provides accurate *E*-value assignments, we are able to offer the option to plot the proportion of false discoveries (PFD) for both peptides and proteins via the Sorić formula [15]. To plot these graphs, one first selects the Plot Results/Plot PFD option in the main menu, then selects one of the three file types (protein PFD, protein cluster PFD, peptide PFD), and then navigates to the result file location and clicks open to generate the PFD plot. Figure 1d displays the peptide identification PFD plot of this example run.

To show that RAId performs comparably to other search tools, we analyze S-1, S-2 and S-3, the triplicate of UPS-1, using RAId_GUI (v.Dec.7.2017), SEQUEST HT [16] (v.1.1), and Mascot (v.2.5.1, http://www.matrixscience.com/help.html) with the same search parameters. Proteome Discoverer (v1.4, http://www.thermofisher.com/en/home.html) is employed for protein ID when SEQUEST and Mascot are used. For each method, its identification results for the triplicate samples (S-1, S-2, and S-3) are displayed in terms of the number of identified proteins that are true positives (TP) alongside with the number of identified proteins that are false positives (FP). For SEQUEST: S-1 21(TP) & 1(FP), S-2 23(TP) & 5(FP), S-3 12(TP) & 1(FP); for Mascot: S-1 27(TP) & 0(FP), S-2 16(TP) & 0(FP), S-3 20(TP) & 0(FP); and for RAId using RAId_GUI with cutoff *E*-value 1: S-1 32(TP) & 1(FP), S-2 33(TP) & 2(FP), S-3 31(TP) & 0(FP). The lists of identified proteins are provided in Additional file 1. More elaborate performance assessment of RAId in comparison with other methods can be found in [11].

### Additional tools

To facilitate further data analyses, we included in the RAId_GUI package three additional tools, which we briefly summarize.

#### Spectra viewer

Users may visualize the experimental data files by selecting the Tools/View MS spectrum option in the main menu. The viewer window starts with displaying the peptide chromatography plot with retention time along the abscissa and the normalized total MS^1^ ion current along the ordinate. The user can select to view either all MS^1^ peaks or only MS^1^ peaks that were selected for further fragmentations. We call the former the MS level and the latter the MS/MS (MS^2^) level. Figure 3a displays the chromatogram of the example data. When the user selects a peak on the chromatogram, two new windows appear. At the MS^1^ level, the first new window displays all m/z peaks at the selected retention time while the second window shows the same m/z peaks deconvoluted to charge one. At the MS/MS level, the first new window displays the MS/MS fragments recorded in the data file while the second window shows the peaks processed by the filtering function (RAId score, Hyperscore, Kscore or XCorr) chosen under the Tools/View MS spectrum option. For the current example, RAId score filtering function is selected.

The user can zoom in by selecting on a graph a segment of abscissa with the left mouse key pressed, and zoom out by pressing the right mouse key. Clicking on the Show data button yields a vertical table with values of peaks on the corresponding graph. The graph can be saved in either png or pdf formats by pressing the Export images button and specifying the output filename. Similarly, the peaks data can by saved to a file in the comma separated text format by clicking the Export data button.

#### MS product tool

The MS product tool can be accessed by selecting the Tools/Peptide Product-Ions option in the main menu. Given a peptide, this tool creates a table of the monoisotopic masses of the peptide’s fragments. When a corresponding spectrum file is also provided, this tool searches for experimental evidences in the spectrum and allows users to visualize these peaks (with annotations) on a graph. Figure 3b is an example taken from the peptide FDEFFSEGCAPGSK belonging to protein P02787 of Fig. 2. P02787 is the top ranking (highlighted) protein in Fig. 2, while FDEFFSEGCAPGSK is the peptide of record number 19 belonging to P02787. The interface and functionality are similar to that of the spectra viewer tool. The table and the graph can be saved to files by pressing corresponding buttons.

#### MIDAs

The third tool is for Molecular Isotopic Distribution Analysis (MIDAs) [17]. This tool is a useful add-on when studying proteomics as it calculates swiftly and displays the theoretical isotopic distributions for the input molecules, providing additional signature for each peptide. In particular, MIDAs allows for customized isotopic abundances and thus might be useful for analyzing data from customized experiments. The default isotopic abundances are obtained from [18, 19].

## Conclusions

In this manuscript, we describe some important features of the RAId_GUI, which is user-friendly and versatile. Once search parameters are selected and experimental data specified, without needing further interventions the suite analyzes the data all the way to reporting identified peptides and then proteins with accurate statistical significance assignments.

## Limitations

Written in Java, the GUI can be launched in any platform. However, the RAId code package utilizes several functions such as parallelization and shared memory that are specifically programmed in the Linux environment. It is our future plan to enable RAId code to run on more platforms, thus maximizing the accessibility of RAId through its GUI.

## Additional file

**Additional file 1.** The lists of identified proteins by various search engines. In this excel file, the readers may find the proteins identified by different search tools listed in the main text while using UPS-1 protein mixture triplicate samples S-1, S-2, and S-3. In the last sheet of this file contains the accession numbers of proteins in UPS-1.

## Abbreviations

RAId: Robust Accurate Identification software package
MIDAs: Molecular Isotopic Distribution Analysis tool
GUI: graphical user interface
CLT: central limit theorem
EVD: extreme value distribution
APP: all possible peptides
PFD: proportion of false discoveries
